# Preventive service use among Medicaid expansion adults, 2014–2019

**DOI:** 10.1093/haschl/qxag117

**Published:** 2026-05-15

**Authors:** Michelle Bronsard, Sarah Hirsch, Dawn Pepin, Terrika Barham, Angela T Estadt, Michelle Van Handel, Joshua A Salomon, Adrienne Sabety

**Affiliations:** Department of Health Policy, Stanford University, Stanford, CA 94305, United States; Department of Health Policy, Stanford University, Stanford, CA 94305, United States; Office of the Director, National Center for HIV, Viral Hepatitis, STD, and TB Prevention, Centers for Disease Control and Prevention, Atlanta, GA 30329, United States; Office of Health Equity, National Center for HIV, Viral Hepatitis, STD, and TB Prevention, Centers for Disease Control and Prevention, Atlanta, GA 30329, United States; Office of the Director, National Center for HIV, Viral Hepatitis, STD, and TB Prevention, Centers for Disease Control and Prevention, Atlanta, GA 30329, United States; Office of the Director, National Center for HIV, Viral Hepatitis, STD, and TB Prevention, Centers for Disease Control and Prevention, Atlanta, GA 30329, United States; Department of Health Policy, Stanford University, Stanford, CA 94305, United States; Department of Health Policy, Stanford University, Stanford, CA 94305, United States

## Abstract

**Introduction:**

In 2014, the Affordable Care Act (ACA) mandated that newly eligible Medicaid expansion beneficiaries receive recommended preventive services at no cost. Yet, evidence regarding the Medicaid expansion population's use of preventive services is limited.

**Methods:**

Medicaid claims data from 2011 to 2019. We focused on 12 selected preventive services that received new, revised, or upheld A or B USPSTF ratings since 2010.

**Results:**

We find that receipt of preventive services among the Medicaid Expansion population increased from 22% in 2014 to 26% in 2019, with 41% of beneficiaries receiving at least one of the 12 examined services from 2014 to 2019. The most received services were screening tests for HIV infection, cervical cancer, chlamydia and gonorrhea, and hepatitis C virus infection. Receipt of at least one preventive service was highest among non-Hispanic Asian, non-Hispanic Black, and Hispanic beneficiaries.

**Conclusion:**

Future changes to Medicaid expansion eligibility or preventive services recommendations could potentially disrupt these utilization patterns, with possible disproportionate effects on people from racial and ethnic minority populations.

## Introduction

Implemented in 2014, the Patient Protection and Affordable Care Act (ACA) incentivized states to expand Medicaid eligibility to nondisabled, nonpregnant adults with incomes up to 138% of the federal poverty level (“Medicaid expansion”). The ACA also mandated that Medicaid expansion beneficiaries receive services recommended by the US Preventive Services Task Force (USPSTF) with an A or B rating at zero out-of-pocket cost.^1^

Describing patterns of preventive service use among Medicaid expansion beneficiaries provides important context for the ACA's coverage and cost-sharing provisions, their potential impact on access to care, and how these provisions may impact the use of preventive care. Preventive services are beneficial and cost-effective for low-income adults, a population that faces a high burden of preventable chronic disease and premature mortality. For instance, increased use of preventive services reduces complications and premature deaths from cancer and cardiovascular disease.^2,3^ This may be especially true for populations identifying as racial and ethnic minorities who face disproportionate burdens of the conditions these preventive services address and have historically faced greater barriers to accessing preventive care.^4^ However, evidence on preventive service use among Medicaid expansion beneficiaries remains mixed, likely due to a reliance on small samples and survey data.^5-7^

We analyzed Medicaid claims data across the 31 states and the District of Columbia, which expanded Medicaid from 2014 to 2019. We focused on 12 selected preventive services receiving new, revised, or upheld A or B USPSTF ratings since 2010. We examined trends in the receipt of preventive services among Medicaid expansion beneficiaries by age, sex, region, and race and ethnicity.

## Study data and methods

We used Medicaid claims data from 2011 to 2019, consisting of Medicaid Analytic eXtract Files (2011-2014) and Transformed Medicaid Statistical Information System Analytic Files (2015-2019), and analyzed the data in Redivis (RRID:SCR_023111).^8^ We aggregated individuals across geography and time using beneficiary identifiers. We categorized beneficiary age, state of residence, biological sex, self-reported race and ethnicity, annual enrollment status, and eligibility type using the demographic file. The study used deidentified secondary data, approved by Stanford University's institutional review board. Generative artificial intelligence tools were used in a limited manner to assist with editing for clarity and grammar.

## Study population

**Figure 1:**
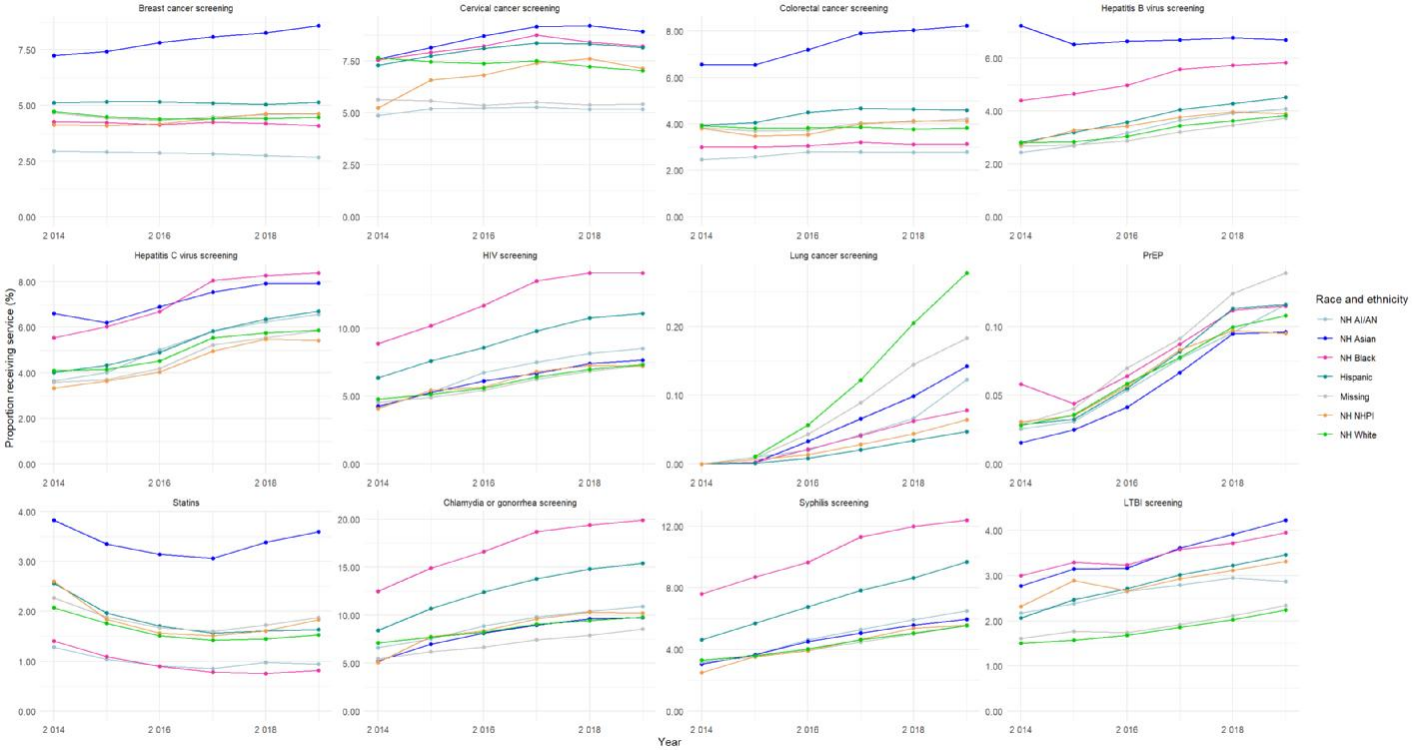
Proportions of Medicaid expansion beneficiaries receiving selected preventive services by race and ethnicity, 2014-2019. Abbreviations: AI, American Indian; AN, Alaska Native; LTBI, latent tuberculosis infection; NH, Non-Hispanic; NHPI, Native Hawaiian and Other Pacific Islander. For each service, rates are calculated as the number of recipients receiving the selected preventive service over the Medicaid expansion population aged 18-64 years in the relevant race and ethnicity category.

The study population included Medicaid expansion beneficiaries aged 18-64 years in the 31 states and the District of Columbia, which expanded Medicaid during 2014-2019. We focused on nondisabled adults with incomes up to 138% of the federal poverty level who were enrolled for at least 1 month. During 2014-2015, we imputed eligibility based on beneficiaries’ subsequent status in late 2015 and early 2016 in the 12 states that misreported eligibility (Supplement A). We defined a beneficiaries’ race and ethnicity as the most frequently reported nonmissing value from 2011 to 2019 (Figure 1).

## Outcomes

We examined 12 preventive services receiving new, revised, or upheld A or B ratings from USPSTF since 2010: statins for primary prevention of cardiovascular disease and preexposure prophylaxis for HIV, and screening tests for breast cancer, lung cancer, colorectal cancer, cervical cancer, latent tuberculosis infection, hepatitis C virus infection, hepatitis B virus infection, HIV infection, chlamydia and gonorrhea, and syphilis infection. We identified claims for each service using Current Procedural Terminology and Healthcare Common Procedure Coding System codes from inpatient, outpatient, and prescription claims (Table S1 Current Procedural Terminology codes were used to identify persons receiving services. https://www.ama-assn.org/practice-management/cpt; Healthcare Common Procedure Coding System codes were used to identify persons receiving services. https://www.cms.gov/Medicare/Coding/MedHCPCSGenInfo*. International Classifications of Diseases, Ninth Revision* (ICD-9) and *Tenth Revision* (ICD-10) were used to identify persons with an exclusionary diagnosis.

## Statistical analysis

We calculated the proportion of beneficiaries who received 1 or more preventive services during 2014-2019. We also calculated an annual service use proportion: the number of beneficiaries receiving 1 or more services in a year divided by the number of beneficiaries enrolled for at least 1 month that year. A beneficiary was defined as eligible for a service if they met the inclusion criteria specified by the USPSTF. A beneficiary was defined as receiving a service if (1) we observed a claim with a procedure code indicating the service and (2) the beneficiary satisfied the inclusion criteria specified by the USPSTF (see Tables S1 and S2).

## Limitations

There are several limitations. First, we are unable to determine why beneficiaries receive preventive services. Guidelines for some services changed over the study period, which could have influenced whether beneficiaries received those services. Second, we developed the exclusion and inclusion criteria for the numerator to balance sensitivity and specificity in identifying service use; the approach does not precisely capture the proportion of individuals with up-to-date screenings. Third, while we categorize beneficiaries’ receipt of service, we do not determine whether beneficiaries received subsequent downstream care. Fourth, this is a descriptive study of service utilization patterns among Medicaid expansion beneficiaries. We do not make causal claims about the effect of Medicaid expansion itself, as we do not compare expansion states to non-expansion states or use quasi-experimental methods to isolate the impact of the policy change. Fifth, we cannot confirm what beneficiaries actually paid due to incomplete out-of-pocket data in the Medicaid claims. Sixth, the 9.8% of beneficiaries with missing race and ethnicity from 2011 to 2019 were directly categorized as “missing” race. While T-MSIS race and ethnicity data quality varied across states, we use the most frequently reported non-missing value from 2011 to 2019 to mitigate potential misclassification. Additionally, our large sample size provides robust estimates even after excluding those with missing data. Seventh, we use a beneficiary's prior claims to identify whether they met the inclusion criteria specified by the USPSTF. However, we do not observe pre-expansion claims for 63% of our study population and we assume a beneficiary meets our inclusion criteria unless we observe otherwise.

## Study results

The study population included 29 600 366 Medicaid expansion beneficiaries from 2014 to 2019 (Table 1). Within Medicaid expansion beneficiaries, 15 437 253 were women (52%), and 13 220 392 self-identified as members of a racial or ethnic minority group (45%).

**Table 1:** Medicaid expansion beneficiaries by selected characteristics, 2014-2019.

Year	2014		2015		2016		2017		2018		2019		2014-2019^a^	
	*n*	%	*n*	%	*n*	%	*n*	%	*n*	%	*n*	%	*n*	%
Unique beneficiaries	8 490 997		13 186 580		15 455 036		16 496 851		16 317 538		16 463 384		29 600 366	
By race and ethnicity														
Hispanic	1 743 264	20.5	2 696 307	20.4	3 230 433	20.9	3 476 214	21.1	3 453 835	21.2	3 399 131	20.6	5 978 553	20.2
NH Asian	549 650	6.5	817 296	6.2	960 879	6.2	992 303	6.0	971 200	6.0	964 673	5.9	1 723 740	5.8
NH AI/AN	102 087	1.2	154 217	1.2	197 932	1.3	218 911	1.3	225 688	1.4	231 018	1.4	380 101	1.3
NH Black	1 312 860	15.5	2 031 745	15.4	2 476 636	16.0	2 765 012	16.8	2 804 076	17.2	2 919 166	17.7	4 849 639	16.4
NH NHPI	107 335	1.3	147 305	1.1	147 949	1.0	130 143	0.8	123 533	0.8	124 381	0.8	288 359	1.0
NH White	3 859 219	45.5	6 121 702	46.4	7 093 399	45.9	7 434 385	45.1	7 271 526	44.6	7 350 695	44.6	13 252 966	44.8
Missing	736 757	8.7	1 092 905	8.3	1 274 675	8.2	1 413 104	8.6	1 408 248	8.6	1 419 005	8.6	2 912 996	9.8
By sex														
Men	4 200 397	49.5	6 484 559	49.2	7 628 181	49.4	8 043 448	48.8	7 937 536	48.6	7 950 767	48.3	14 196 416	48.0
Women	4 290 600	50.5	6 702 021	50.8	7 826 855	50.6	8 453 403	51.2	8 380 002	51.4	8 512 617	51.7	15 437 253	52.2
By age^b^														
18—29	2 780 766	32.7	4 552 954	34.5	5 406 293	35.0	5 827 903	35.3	5 726 712	35.1	5 681 725	34.5	11 839 985	40.0
30—39	1 692 353	19.9	2 806 670	21.3	3 373 213	21.8	3 676 700	22.3	3 711 236	22.7	3 811 138	23.1	6 061 675	20.5
40—49	1 519 899	17.9	2 269 912	17.2	2 598 974	16.8	2 742 384	16.6	2 708 688	16.6	2 749 020	16.7	4 756 347	16.1
50—59	1 820 078	21.4	2 567 168	19.5	2 909 545	18.8	2 995 154	18.2	2 907 467	17.8	2 915 156	17.7	4 933 288	16.7
60—64	677 901	8.0	989 876	7.5	1 167 011	7.6	1 254 710	7.6	1 263 435	7.7	1 306 345	7.9	2 009 071	6.8
By region														
Northeast	1 088 419	12.8	2 300 272	17.4	2 625 261	17.0	2 730 229	16.6	2 735 813	16.8	2 750 998	16.7	5 178 555	17.5
Midwest	1 765 454	20.8	2 845 772	21.6	3 454 359	22.4	4 014 086	24.3	4 011 958	24.6	3 854 769	23.4	6 901 050	23.3
South	1 159 674	13.7	1 744 955	13.2	2 072 757	13.4	2 328 507	14.1	2 333 356	14.3	2 790 247	16.9	4 631 728	15.6
West	4 477 450	52.7	6 295 581	47.7	7 302 659	47.3	7 424 029	45.0	7 236 411	44.3	7 067 370	42.9	13 139 324	44.4

Abbreviations: AI, American Indian; AN, Alaska Native; NH, Non-Hispanic; NHPI, Native Hawaiian and Other Pacific Islander.

^a^All individuals enrolled in Medicaid expansion for at least 1 month between 2014 and 2019.

^b^For aggregate years, age is measured at first entry in the dataset.

Table 2 reports the numbers and proportions of Medicaid beneficiaries overall and within specific demographic strata receiving 1 or more services by year. A total of 12 230 477 beneficiaries received 1 or more examined preventive services during 2014-2019 (41% = 12 230 477/29 600 366), including 56% of women and 26% of men. Yearly proportions of beneficiaries receiving at least 1 examined service ranged from 1 850 806 in 2014 (22%) to 4 351 939 in 2019 (26%). Across 2014-2019, yearly proportions ranged from 32% to 38% among women and 11% to 14% among men.

**Table 2:** Medicaid expansion beneficiaries receiving 1 or more selected preventive services,^a^ 2014-2019.

Year	2014		2015		2016		2017		2018		2019		2014-2019^b^	
	*n*	%	*n*	%	*n*	%	*n*	%	*n*	%	*n*	%	*n*	%
Received ≥ 1 service^c^	1 850 806	21.8	2 989 705	22.7	3 646 734	23.6	4 168 087	25.3	4 221 085	25.9	4 351 939	26.4	12 230 477	41.3
By race and ethnicity														
Hispanic	384 943	22.1	652 807	24.2	827 907	25.6	947 557	27.3	974 752	28.2	985 183	29.0	2 665 546	44.6
NH Asian	143 324	26.1	226 820	27.8	279 300	29.1	305 256	30.8	309 287	31.8	313 647	32.5	836 247	48.5
NH AI/AN	17 220	16.9	27 851	18.1	39 024	19.7	45 643	20.9	47 845	21.2	49 756	21.5	140 611	37.0
NH Black	321 225	24.5	537 121	26.4	689 602	27.8	838 379	30.3	856 539	30.5	898 710	30.8	2 300 202	47.4
NH NHPI	19 734	18.4	30 759	20.9	31 570	21.3	30 749	23.6	30 564	24.7	30 830	24.8	108 555	37.6
NH White	809 565	21.0	1 302 370	21.3	1 531 996	21.6	1 704 368	22.9	1 695 303	23.3	1 753 677	23.9	5 207 503	39.3
Missing	135 473	18.4	203 514	18.6	240 635	18.9	287 371	20.3	297 793	21.1	311 014	21.9	927 907	31.9
By sex														
Men	474 363	11.3	738 623	11.4	913 970	12.0	1 059 661	13.2	1 093 783	13.8	1 148 619	14.4	3 656 738	25.8
Women	1 376 443	32.1	2 251 082	33.6	2 732 764	34.9	3 108 426	36.8	3 127 302	37.3	3 203 320	37.6	8 577 957	55.6
By age^d^														
18 to 29	504 649	18.1	930 588	20.4	1 172 889	21.7	1 360 532	23.3	1 386 605	24.2	1 412 424	24.9	4 303 800	36.3
30 to 39	285 485	16.9	485 834	17.3	618 071	18.3	734 088	20.0	766 187	20.6	816 019	21.4	2 216 205	36.6
40 to 49	347 244	22.8	518 548	22.8	603 074	23.2	666 878	24.3	667 400	24.6	691 746	25.2	2 008 384	42.2
50 to 59	523 602	28.8	764 297	29.8	898 157	30.9	993 965	33.2	974 840	33.5	986 741	33.8	2 620 027	53.1
60 to 64	202 225	29.8	311 318	31.5	380 303	32.6	444 302	35.4	458 923	36.3	480 212	36.8	1 082 061	53.9
By region														
Northeast	317 463	29.2	601 258	26.1	670 877	25.6	712 631	26.1	724 171	26.5	778 324	28.3	2 192 028	42.3
Midwest	392 361	22.2	686 743	24.1	837 907	24.3	1 053 757	26.3	1 049 403	26.2	1 036 657	26.9	2 931 040	42.5
South	246 934	21.3	304 600	17.5	430 233	20.8	546 821	23.5	568 243	24.4	671 386	24.1	1 663 803	35.9
West	894 048	20.0	1 397 104	22.2	1 707 717	23.4	1 854 878	25.0	1 879 268	26.0	1 865 572	26.4	5 492 477	41.8
Received selected service^e^														
Breast cancer screening tests	414 307	4.9	622 235	4.7	723 477	4.7	780 210	4.7	771 229	4.7	784 846	4.8	2 488 864	8.4
Cervical cancer screening tests	623 416	7.3	974 095	7.4	1 163 068	7.5	1 282 675	7.8	1 237 716	7.6	1 220 044	7.4	4 854 303	16.4
Colorectal cancer screening tests	334 307	3.9	507 236	3.9	620 416	4.0	687 267	4.2	670 470	4.1	679 809	4.1	2 308 944	7.8
Hepatitis B virus infection screening tests	282 272	3.3	448 144	3.4	568 286	3.7	677 306	4.1	702 830	4.3	740 007	4.5	2 921 375	9.9
Hepatitis C virus infection screening tests	374 857	4.4	598 092	4.5	782 122	5.1	1 007 766	6.1	1 047 957	6.4	1 089 467	6.6	4 083 644	13.8
HIV infection screening tests	482 059	5.7	842 841	6.4	1 118 090	7.2	1 374 421	8.3	1 475 832	9.0	1 539 233	9.3	5 251 429	17.7
Lung cancer screening tests	<16	0.0	898	0.0	5 755	0.0	13 033	0.1	21 067	0.1	28 731	0.2	60 362	0.2
Preexposure prophylaxis for HIV (PrEP)	2720	0.0	4735	0.0	9024	0.1	13 284	0.1	17 315	0.1	18 571	0.1	40 424	0.1
Statin use for primary prevention of cardiovascular disease	186 513	2.2	235 997	1.8	240 392	1.6	238 689	1.4	244 027	1.5	258 539	1.6	773 453	2.6
Chlamydia and gonorrhea screening tests	672 840	7.9	1 216 684	9.2	1 588 640	10.3	1 902 685	11.5	1 986 315	12.2	2 082 219	12.6	4 825 907	16.3
Syphilis infection screening tests	358 062	4.2	631 809	4.8	853 551	5.5	1 063 371	6.5	1 150 977	7.0	1 262 309	7.7	4 054 542	13.7
Latent tuberculosis infection screening tests	166 647	2.0	283 076	2.1	349 333	2.3	415 724	2.5	442 023	2.7	482 844	2.9	1 785 743	6.0

Abbreviations: AI, American Indian; AN, Alaska Native; LTBI, latent tuberculosis infection; NH, Non-Hispanic; NHPI, Native Hawaiian and Other Pacific Islander; PrEP, preexposure prophylaxis.

^a^Services included are statins, PrEP for HIV, and screening tests for breast cancer, lung cancer, colorectal cancer, cervical cancer, latent tuberculosis infection, hepatitis C virus infection, hepatitis B virus infection, HIV infection, chlamydia and gonorrhea, and syphilis infection.

^b^For aggregate years, proportions (%) are calculated as the number of beneficiaries receiving at least 1 service during any year between 2014 and 2019 divided by the number of unique Medicaid Expansion beneficiaries in the dataset over the period, overall or within specific demographic strata.

^c^For each demographic category (race and ethnicity, sex, age, region), proportions (%) are calculated as the number of service recipients in that category divided by the Medicaid expansion population in that category (reported in Table 1).

^d^Age categories are assigned based on the age at first appearance in the dataset.

^e^For specific services in this section of the table, proportions (%) are calculated as the number of persons receiving that service divided by the total number of unique Medicaid expansion beneficiaries in the sample during that year (reported in Table 1, Row 1).

Table 2 shows that receipt of at least one preventive service was highest among non-Hispanic Asian (49%), non-Hispanic Black (47%), and Hispanic (45%) beneficiaries. During 2014-2019, the greatest relative changes in service receipt were among non-Hispanic Native Hawaiian and Other Pacific Islander (35%), Hispanic (31%), and non-Hispanic American Indian or Alaska Native (28%) beneficiaries.

Beneficiaries received the highest proportions of screening tests for HIV infection (18%), cervical cancer (16%), chlamydia and gonorrhea (16%), and hepatitis C virus infection (14%) (Table 2). The receipt of specific services also varied by race and ethnicity (Figure 1 and Table 2). Compared with other groups, non-Hispanic Black and Hispanic beneficiaries received screening tests for HIV infection, syphilis infection, and chlamydia and gonorrhea in the highest proportions. Screening proportions for breast cancer, colorectal cancer, and hepatitis B virus infection were highest among non-Hispanic Asian beneficiaries. Non-Hispanic White beneficiaries had the highest lung cancer screening proportions.

## Discussion

During 2014-2019, 41% of Medicaid expansion beneficiaries received at least one preventive service covered under the ACA preventive services requirement. Service receipt was highest among non-Hispanic Asian, non-Hispanic Black, and Hispanic beneficiaries, consistent with studies focused on HIV testing or using survey data.^9,10^

A recent legal challenge to the ACA preventive services requirement, *Kennedy v. Braidwood Mgt.*, raised concerns about the continued protection of no-cost coverage of USPSTF-recommended services.^11,12^ Although the case eventually was resolved in favor of continuing the mandate, the potential for future challenges to the ACA remains. This issue is of particular concern because research shows that increases in out-of-pocket costs lead to a decline in the use of preventive services.^13^ For instance, a modest increase in the cost of preexposure prophylaxis for HIV was associated with patients abandoning their prescriptions.^14^ Together with our descriptive results, these findings suggest that changes to Medicaid and USPSTF guidance or coverage requirements could impact the use of preventive services, widening existing health inequities in the United States.

Taken together, these findings indicate that a substantial proportion of Medicaid expansion beneficiaries received one of the 12 examined preventive services. Our observed trends show increasing use of these services from 2014 to 2019. While our descriptive study cannot establish causality, changes to insurance coverage or the requirement could potentially disrupt these utilization patterns, with possible disproportionate effects on people from racial and ethnic minority populations.

## Supplementary Material

qxag117_Supplementary_Data
