# Supplementary material for: Preventive service use among Medicaid expansion adults, 2014–2019
Source: Health Aff Sch. 2026 May 15;4(6):qxag117. doi: 10.1093/haschl/qxag117 (PMC13234943; doi:10.1093/haschl/qxag117)
Supplement: qxag117_Supplementary_Data [file qxag117_supplementary_data.zip › Supplementary file.docx]

### Supplemental Content

**A. Timeline of Medicaid expansion**

Between 2014 and 2019, 31 states and the District of Columbia (DC) adopted Medicaid expansion. Of these, 24 states and DC implemented the expansion in 2014. Alaska, Indiana, and Pennsylvania expanded in 2015, Louisiana and Montana in 2016, and Virginia and Maine in 2019. Two states (Vermont and Massachusetts) had expanded before 2010. At the start of 2020, 17 states had not yet expanded, including the following states: Alabama, Florida, Georgia, Idaho, Kansas, Mississippi, Missouri, Nebraska, North Carolina, Oklahoma, South Carolina, South Dakota, Tennessee, Texas, Utah, Wisconsin, and Wyoming.

**Capturing Medicaid expansion beneficiaries**

To restrict the sample to Medicaid expansion beneficiaries, we identified and aggregated beneficiaries denoted in the data files by a monthly group eligibility code of ‘72’, or ‘Adult Group — Individuals at or below 133% FPL [federal poverty level], [aged]18-64, newly eligible for all states’. We also included the following beneficiaries who were incorrectly coded:

- Newly eligible beneficiaries who had been mistakenly coded, as documented in the Data Quality Atlas and Transformed Medicaid Statistical Information System Analytic Files technical briefs
- Newly eligible beneficiaries residing in states that expanded Medicaid initially through waivers for particular groups, such as the Section 1115 demonstration waiver (Arizona, Arkansas, Iowa, Indiana, Kentucky, Michigan, Montana, New Hampshire, and Pennsylvania)

For nearly all states with miscoded beneficiaries, miscoding occurred only in 2014 and 2015. For these beneficiaries, we imputed their eligibility data based on their subsequent status in late 2015 and early 2016. Two states, Vermont and Massachusetts, had no newly eligible beneficiaries because these states expanded before 2010. For North Dakota, eligibility data was unreliable in earlier years, so we only included beneficiaries enrolled after 2016. To validate the inclusion of these beneficiaries, we cross-checked the numbers we obtained against the official counts of newly eligible beneficiaries in each state, month, and year published in the CMS Open Data Table.

**Supplement Table 1.** **Procedure, Diagnosis, and Drug Codes for Selected Preventive Services**

| **Service** | **Codes to identify service recipients** | | **Codes to identify excluded individuals^a^** | | **Criteria for counting as service recipients for this service** |
| --- | --- | --- | --- | --- | --- |
|  | **Code type** | **Code number** | **Code type** | **Code number** |  |
| Breast cancer screening test | CPT | 76083, 76085, 76092, 77052, 77057, 77063, 77067 | ICD-10 | Z15.01, Z85.3 | Women aged 40-64y without a code indicating a history of breast cancer or genetic risk factors |
|  | HCPCS | G0202, G0203 | ICD-9 | V10.3, V84.01 |  |
| Cervical cancer screening test | CPT | 87620-87622, 87624, 87625, 88141-88143, 88147, 88148, 88150, 88152, 88153, 88164-88167, 88174, 88175, 0500T | CPT | 57530, 57531, 57540, 57545, 57550, 57555, 57556, 58150, 58152, 58200, 58210, 58240, 58260, 58262, 58263, 58267, 58270, 58275, 58280, 58285, 58290-58294, 58548, 58550, 58552-58554, 58570-58573, 58575, 58951, 58953, 58954, 59856, 59135 | Women aged 21-64y without a code indicating a history of cervical cancer or no cervix |
|  | HCPCS | G0101, G0123, G0124, G0141, G0143, G0144, G0145, G0147, G0148, 0500T, P3000, P3001, Q0091, G0476 |  |  |  |
|  | ICD-10 | Z11.51 | ICD-10 | 0UTC0ZZ, 0UTC4ZZ, 0UTC7ZZ, 0UTC8ZZ, Q51.5, Z90.710, Z90.712, C53.X |  |
|  | ICD-9 | V73.81 | ICD-9 | 75243, 674, 180.X, V88.03, V88.01 |  |
| Colorectal cancer screening test | CPT | 45330, 45331, 45338, 45346, 45378, 45380, 45381, 45384, 45385, 45388, 74261-74263, 81528, 82270, 82274 | ICD-10 | C18.X, C19.X, C20.X, C78.5, Z85.038, Z85.048 | Individuals aged 45-64y without a code indicating a history of colorectal cancer |
|  | HCPCS | G0104, G0105, G0107, G0121, G0328 | ICD-9 | 153.X, 154.0, 154.1, 197.5, V10.05, V10.06 |  |
| Hepatitis B virus infection screening test | CPT | 86704, 86706, 87340, 87341, 87515, 87517 | CPT | 90636, 90697, 90723, 90739, 90740, 90743, 90745-90748, 90759 | Individuals aged 18-64y without a code indicating a history of acute or chronic hepatitis B or hepatitis B vaccination |
|  | HCPCS | G0499 | HCPCS | 4149F, 4157F, 4275F, G0010, G8870, M0201 |  |
|  |  |  | ICD-10 | B16.X, B17.0, B18.0, B18.1, B19.1X |  |
|  |  |  | ICD-9 | 070.2X, 070.3X |  |
| Hepatitis C virus infection screening test | CPT | 80074, 86803, 86804, 87520, 87521, 87522, 87902 | ICD-10 | B17.1X, B18.2, B19.2X, K70.1X | Individuals aged 18-64y without a code indicating a history of acute or chronic hepatitis C |
|  | HCPCS | G0472, 3266F | ICD-9 | V02.62, 070.4X, 070.5X, 070.7X |  |
| HIV infection screening test | CPT | 86689, 86701-86703, 87389-87391, 87534-87539, 87806 | ICD-10 | B20.X, B21.X, B22.X, B23.X, B24, B97.35, Z21 | Individuals aged 18-64y without a code indicating a history of HIV |
|  | HCPCS | G0432, G0433, G0435, G0437, G0298, G0475, S3645 |  |  |  |
|  | ICD-10 | Z11.4 | ICD-9 | 042, 043, 044, 07953, V08 |  |
| Lung cancer screening test | CPT | 71271 | ICD-10 | Z86.005, C34.9X, Z85.1X | Individuals aged 50-64y without a code indicating a history of lung cancer |
|  | HCPCS | G0927, S8032 | ICD-9 | 162.X, V10.11 |  |
| Pre-exposure prophylaxis for HIV (PrEP) | NDC | 00093760756, 00093770456, 00378193093, 00904717207, 16714053401, 31722056030, 33342010607, 35356007003, 35356007006, 35356007030, 42291043930, 42385095330, 42543071904, 49702026423, 50090087000, 50090087002, 50090087003, 50090595701, 50436070101, 51407011230, 52959096903, 54569558800, 54569558802, 54569558803, 54868514100, 55045348103, 60505420203, 61919066902, 61958070101, 61958070301, 61958070401, 61958200201, 61958200202, 64380071904, 65862035430, 66336003203, 67263026030, 68071211203, 68180028706, 68258198303, 69097020902, 69097074102, 69238209503, 70518023000, 70710136703, 72189022702, 72189031203, 76282067730 | ICD-10 | B18.0, B18.1, B19.1, B20.X, B21.X, B22.X, B23.X, B24, B97.35, Z21 | Individuals aged 15-64 yrs with two or more consecutive PrEP prescriptions and without a code indicating a history of HIV or chronic HBV^b^ |
|  | ICD-10 | Z29.81 | ICD-9 | 042, 043, 044, 07953, V08, 070.22, 070.23, 070.32, 070.33 |  |
| Statin use for primary prevention of cardiovascular disease | NDC | Multiple NDCs (2118) | ICD-10 | I10.X, I11.X, I12.X, I13.X, I14.X, I15.X, I20.X, I21.X, I22.X, I23.X, I24.X, I25.X, I50.X, I60.X, I61.X, I62.X, 163.X, 165.X, 166.X | Individuals aged 40-64y without a code indicating a history of cardiovascular disease or familial hypercholesterolemia |
|  |  |  | ICD-9 | 401.X-405.X, 410.X-414.X, 428.X, 430.X-434.X |  |
| Syphilis infection screening test | CPT | 86592, 86593, 86780, 87285, 1011977, 0064U, 0065U, 0210U |  | | Individuals aged 18-64y |
|  | HCPCS | 3512F, G9228, G9229, G0450 |  |  |  |
| Latent tuberculosis infection screening test | CPT | 86480, 86481, 86580, 86585 | ICD-10 | Z86.11, A15.X-A19.X, R76.11, R76.12, J65, P37.0, B90.X | Individuals aged 18-64y without a code indicating a history of tuberculosis disease |
|  | HCPCS | M1003, G9359 |  |  |  |
|  | ICD-10 | Z11.1, Z11.7 | ICD-9 | V12.01, 010.X-019.X |  |
|  | ICD-9 | V74.1, 795.5X |  |  |  |
| Chlamydia and gonorrhea screening test | CPT | 0353U, 87491, 87492, 87591 |  | | Women aged 18-64y |

Abbreviations: CPT, Current Procedural Terminology; HCPCS, Healthcare Common Procedure Coding System; ICD-9, International Classifications of Diseases, Ninth Revision; ICD-10, International Classifications of Diseases, Tenth Revision; NDC, National Drug Code.

^a^ For each service, a beneficiary was considered a non-recipient of the service if any exclusionary code was observed before the time of service receipt.

^b^ A prescription was considered consecutive if it was refilled within 10 days after the end date of the previous prescription. History of chronic HBV was an exclusion criterion to avoid false-positive identification of PrEP for HIV recipients where tenofovir used to treat HBV infection.

**Supplement Table 2. Selected Preventive Services with US Preventive Services Task Force (USPSTF) Grade A or B Recommendations^a^**

| **Service** | **USPSTF Recommendations** | | |
| --- | --- | --- | --- |
|  | **Year** | **Age** | **Population** |
| Breast cancer screening | 2009 | 50-74y | All women |
|  | 2016 | 50-74y |  |
|  | 2024 | 40-74y |  |
| Cervical cancer screening | 2003 | Adults | All women |
|  | 2012 | 21-65y |  |
|  | 2018 | 21-65y |  |
| Chlamydia and gonorrhea screening | 2007 | Adolescents and adults^b^ (chlamydia only) | Women at risk: women who have a new sex partner, more than 1 sex partner, a sex partner with concurrent partners, or a sex partner who has an STI; practice inconsistent condom use when not in a mutually monogamous relationship; or have a previous or coexisting STI. |
|  | 2014 | Adolescents and adults^b^ |  |
|  | 2021 | Adolescents and adults^b^ |  |
| Colorectal cancer screening | 2008 | 50-75y | All |
|  | 2016 | 50-75y |  |
|  | 2021 | 45-75y |  |
| Hepatitis B virus infection screening | 2004 | Recommended against |  |
|  | 2014 | Adolescents and adults | Persons at risk: persons who have injected drugs in the past or currently; MSM; persons with HIV; and sex partners, needle-sharing contacts, and household contacts of persons with hepatitis B. |
|  | 2020 | Adolescents and adults |  |
| Hepatitis C virus infection screening | 2004 | Recommended against |  |
|  | 2013 | Adolescents and adults | Persons at risk: persons with past or current injection drug use. |
|  | 2020 | 18-79y | All |
| HIV infection screening | 2005 | Adolescents and adults | Persons at risk: MSM, persons having anal intercourse without a condom, having vaginal intercourse without a condom and with more than 1 partner whose HIV status is unknown, exchanging sex for drugs or money, having other sexually transmitted infections or a sex partner with an STI, and having a sex partner who is living with HIV or is in a high-risk category |
|  | 2013 | 15-65y | All |
|  | 2019 | 15-65y |  |
| Latent tuberculosis infection screening | 1996 | Adults | Persons at risk: persons who were born in, or are former residents of, countries with high tuberculosis prevalence and persons who live in, or have lived in, high-risk congregate settings (eg, homeless shelters or correctional facilities). |
|  | 2016 | Adults |  |
|  | 2023 | Adults |  |
| Lung cancer screening | 2004 | Insufficient evidence |  |
|  | 2013 | 55-80y | Persons at risk: persons with a 30 pack-year smoking history and currently smoke or have quit within the past 15 years. |
|  | 2021 | 50-80y | Persons at risk: persons with a 20 pack-year smoking history and currently smoke or have quit within the past 15 years. |
| Syphilis infection screening | 2004 | Adolescents and adults | Persons at risk: MSM, persons with HIV, persons with a history of incarceration or sex work. |
|  | 2016 | Adolescents and adults |  |
|  | 2022 | Adolescents and adults |  |
|  | 2023 | Adolescents and adults |  |
| Pre-exposure prophylaxis for HIV (PrEP) | 2019 | Adolescents and adults | Persons at risk: MSM, PWID, sexually active adults who have engaged in anal or vaginal sex in the past 6 months and have a sexual partner who has HIV, a bacterial sexually transmitted infection in the past 6 months, or a history of inconsistent or no condom use with sex partner(s) whose HIV status is not known. |
|  | 2023 | Adolescents and adults |  |
| Statin use for primary prevention of cardiovascular disease | 2008 | Adults | Persons at risk: persons with an estimated 10-year cardiovascular disease risk of 10% or greater and who smoke or have dyslipidemia, diabetes, or hypertension. |
|  | 2016 | 40-75y |  |
|  | 2022 | 40-75y |  |

Abbreviations: MSM, men who have sex with men; PWID, people who inject drugs; STI, sexually transmitted infection.

^a^ For each service, recommendations shown include the last update before 2011 and all subsequent updates.

^b^ Recommended screening all sexually active women aged 24 years or younger and in women aged 25 years or older who are at increased risk for infection.
